# Whole body and partial body cryotherapies – lessons from human practice and possible application for horses

**DOI:** 10.1186/s12917-018-1679-6

**Published:** 2018-12-12

**Authors:** K. Roszkowska, O. Witkowska-Pilaszewicz, M. Przewozny, A. Cywinska

**Affiliations:** 1 Department of Pathology and Veterinary Diagnostics, Faculty of Veterinary Medicine, Warsaw University of Life Sciences, SGGW, Nowoursynowska 159c, Warsaw, Poland; 2 EQUI VET SERWIS, Wygoda 6, 64-320 Buk, Poland

**Keywords:** Cryotherapy, Horse, Inflammation, Cryosauna

## Abstract

Whole body and partial body cryotherapies (WBC and PBC) have been successfully used in human medicine, and currently also are being proposed in veterinary practice.

In horses, only the partial body cryotherapy provided in cryosauna is considered, due to the technical conditions. These therapies have been dedicated to human patients with rheumatic and inflammatory diseases as well as an assistance during training in athletes. The anti-inflammatory effects have been demonstrated clinically and indicated by the changes in several hematological and immunological parameters, however, various patterns have been described, depending on the protocols and the treating subjects. The numbers of white blood cells and the differential counts either increased or remained unchanged but the cytokine concentrations generally changed towards various anti-inflammatory profiles and the modifications of immunological molecules having paracrine effects have been reported.

In equine practice, local cold therapies have been successfully used, so the therapy in cryosauna, which allows for a much shorter procedure with much lower temperature seems promising.

## Background

Cold therapies (cryotherapies) are well-known and widely used in relieving pain and discomfort as well as for reducing certain pathological conditions, particularly acute and chronic inflammation. Whole body and partial body cryotherapy (WBC and PBC) involve the exposure to extremely low temperatures [1]. Both techniques are widely used in humans and currently are being introduced to veterinary medicine as well. In WBC, the extreme cold (below − 100 °C) is rapidly applied to whole patient’s body for a brief time (usually 2–5 min) in a cryochamber large enough for several individuals to be treated at the same time. In PBC, the body except the head is exposed to the same conditions (temperature below − 100 °C for 2–5 min) in the cryosauna which allows the patient’s head to be kept outside the chamber, and the cold is applied to the rest of the body. Thus, cryosauna is intended for one patient only. WBC and PBC are applied to patients with rheumatic and inflammatory diseases but also to healthy subjects (cryostimulation), mainly elite athletes as an assistance during training season [1] and to prevent injuries and stress lesions [2, 3]. Another option, also listed among cryotherapies is the local cryotherapy including ice pack therapy, cold spray anesthetics or local administration of liquid nitrogen or carbon dioxide vapour. All kinds of cryotherapies are common in human medicine while only local methods are widely performed in veterinary medicine.

The use of WBC and PBC in veterinary medicine is limited due to the necessity for the chambers and therapeutic protocols specially designed for animals. Moreover, the procedures should be preceded by adaptation to be properly tolerated by animals. However, the benefits proved in humans and the effects known from local cold therapies in animals encourage to introduce these ways of cold therapy to veterinary medicine, particularly to equine athletes. In the horses, digital application of low temperatures have been successfully used as a remedy for joint and tendon injuries and other pathologies resulting from inflammation, particularly laminitis, which has been also confirmed in experimental models and described the most often [4–13]. Many studies have shown that in laminitis local cold therapies relieve pain and when early applied may prevent developing the acute form of the disease [8, 11, 13] However, these techniques are time and labour consuming and horse may feel discomfort while wearing boots or sleeves filled with ice for many hours [11]. Taking into account that cryochamber/cryosauna allows much shorter procedure (few minutes only) with much lower temperature, it seems promising, however, precise indications should be determined [11]. Another use of cryochamber/cryosauna may be considered in sport horses. Many studies have shown advantages of cryotherapy in human athletes, so maybe similar results can be expected in horses.

### General effects of cryotherapy

Many studies confirmed that cryotherapy is safe and has the beneficial effects in curing various, particularly orthopedic diseases, promotes faster recovery after traumas, supports intense training in healthy athletes and prevents overtraining symptoms [14–18]. Low temperatures have analgesic action and promote recovery by reducing inflammation due to vascular response, hypometabolism and the changes in the production of pro-inflammatory and anti-inflammatory mediators [1, 19].

Analgesia is believed to result from slowed nerve conduction, inhibiting the transmission of pain signals to the dorsal horn of the spinal cord [20–22]. This phenomenon is observed in humans when the body temperature drops to 27 °C [22] and widely used to treat patients suffering from chronic pain, fibromyalgia, acute injury or arthritic pain [21, 23–26]. It has been demonstrated that cold reduces acetylocholine formation [27] and stimulates the noradrenalin release from sympathetic system [28], which circulates and in turn reaches the spinal cord. This has been suggested as additional mechanism involved in pain relief by cold therapy, as spinal administration of noradrenalin has been proved to alleviate pain in human [1, 29]. It is also considered that cold may act as a counterirritant stimulating central pain pathways that then activate descending inhibitory pathways, blocking pain transmission to the brain [21, 30, 31].

Exposure to rapid cooling and so that dramatic decrease of skin temperature during cryosessions results in adaptive vascular, myocardial and hemodynamic changes. These effects include peripheral vasoconstriction followed by vasodilation, decrease in peripheral blood flow, and an increase in a central blood flow [1, 24, 32]. Vasoconstriction results from sympathetic activation of the α_2_- adrenergic receptors of the vascular wall, subsequent vasodilation occurs due to the cessation of noradrenaline release [19, 24]. Thus, sudden response to cold involves the increase of resistance and quick reduction in peripheral blood flow to prevent heat loss. Soon after exposure, the blood is shifted from innervated cutaneous blood vessels to the microcirculation of the skin [33] and then systemic redistribution of blood is observed, as the blood is displaced to bigger vessels, so that the central flow increases [24]. Reduced peripheral blood flow attenuates the inflammatory response and possibly oedema formation [1].

Inflammatory response is also diminished by hypometabolism [4, 19, 34, 35], resulting from decreasing enzymatic activity, apoptosis and mitochondrial failure [36]. Thus, it prevents cellular damage and swelling [20]. Reduction of inflammatory reaction also contributes to analgesia, as the concentrations of inflammatory mediators that irritate peripheral nociceptors are diminished and pain sensation is reduced [20, 21, 37, 38].

The measurable effects of WBC have been proved in numerous human-based studies showing the changes in physiological and biochemical parameters and the functional status of the cells [15, 16, 18]. The changes have been described usually after series of cryostimulation sessions, however even one treatment can cause differences in clinical and laboratory results [16, 32, 39].

### Changes in inflammatory parameters

Many studies have indicated cryotherapy as affecting inflammatory reaction even expressed in peripheral blood composition, including the numbers of leukocytes, essential for the onset of inflammation. However, various changes in hematological profiles in response to cryosessions have been reported, depending on the subjects and the protocols (Table 1). The total leukocyte counts and differential count have been reported to either increase [2, 16] or transiently increase as a result of rapid mobilization [40], or remain unchanged [3, 40, 41]. The increases have been noted particularly in lymphocyte and monocyte numbers [7, 16, 26, 42]. It is generally said that the impact of extremely low temperatures on the white blood cells counts cannot be unambiguously determined. One can talk about a mobilization which may have a positive effect on the immune mechanism of the body, however, wide variations occur.

**Table 1:** Characterization of changes in hematological parameters and cytokines in human after cryochamber sessions

Reference	Subjects	WBC protocols	Parameters		
			Increased	No difference	Decreased
Mila-Kierzenkowska et al. [39]	18 volleyball players	1 session		TNF-β1	IL-6, IL-1β, TNF-α
Pournot et al. [41]	11 long distance runners	4 sessions	IL-1ra	LEU, IL-10, IL-6, TNF-α	CRP
Banfi et al. [17]	10 rugby Players	5 sessions	IL-10, LEU	LYM, MON	IL-2, IL-8
Lubkowska et al. [16]	15 healthy men	10 sessions	LEU, LYM, NEU, MON, EOS, IL-6		
Gizińska et al. [57]	25 women with rheumatoid arthritis	10 sessions			IL-6, TNF-α
Ziemann et al. [43]	18 healthy men	10 sessions (5 days, twice a day)	IL-10		IL-1β
Ziemann et al. [2]	12 professional tennis players	10 sessions (5 days, twice a day)	LEU, IL-6		TNF-α
Lombardi et al. [3]	27 rugby players	14 sessions (7 days, twice daily)		LEU	RBC, Hb, Ht
Szygula et al. [40]	45 military academy students	10 sessions	LEU		RBC, Hb, Ht, IL-3
		20 sessions	LEU		RBC, Hb, Ht, IL-3
		30 sessions	MON	LEU, RBC, Hb, Ht	IL-3
Lubkowska et al. [45]	15 healthy men	5 sessions	IL-10, IL-6		IL-1α
	15 healthy men	10 sessions	IL-10, IL-6		IL-1α
	15 healthy men	20 sessions	IL-10, IL-6		IL-1α

*LEU* leukocyte counts, *MON* monocyte counts, *LYM* lymphocyte counts, *NEU* netrophile counts, *EOS* eosinophil counts, *RBC* erythrocyte counts, *Hb* hemoglobin concentration, *Ht* hematocrit, *IL* interleukine, *TNF* tumor necrosis factor

Changes in leukocyte numbers and differential counts may be transient and affected by various factors, including the stress, thus it is better to prove the anti-inflammatory effect on the basis of the changes in inflammatory mediators’ profiles, which has been clearly demonstrated as a result of cryosessions. The decrease of inflammation results from either lower production and release of proinflammatory agents or higher production and release of anti-inflammatory factors [39–41, 43–45]. Moreover, changes in the expression of the surface molecules, which may play a crucial role in the inhibition of inflammatory reaction by cold therapy, have been also described [13, 46, 47]. Inflammatory reaction is regulated by the complicated network of mediators, but in the context of cryotherapy, the well known regulators of acute inflammation, such as interleukins (IL): IL-1, IL-2, IL-6, IL-8, IL-10, tumor necrosis factor α (TNF-α) and C-reactive protein (CRP) have been mostly studied [14]. Various anti-inflammatory patterns have been presented, which is not surprising due to the variations in the studies’ design regarding the subjects and the protocols of cryostimulation. Moreover, it has also been postulated that the effect of whole body cryotherapy may be linked to the modifications of immunological molecules having paracrine effects, rather than systemic immune functions [15].

Cryostimulation has been widely tested in healthy athletes as a support for recovery after heavy exercise and in orthopedic patients. Pournot et al. [41] described the changes in inflammatory parameters in well-trained runners up to 96 h after cryosession. The anti-inflammatory pattern has been shown as marked decrease in CRP level (the major acute phase protein in humans) after cryosession when compared to control group undergoing passive recovery. Although the levels of IL-6, IL-10 and TNF- α did not differ between control and cold-treated group, the increase of proinflammatory IL-1β that naturally occurs after damaging exercise was limited in participants after cryosession. Simultaneously, larger increase in cytokine inhibitor IL-1ra occurred. It has been proposed that the mechanism of this phenomenon involved the stimulation of β-adrenoreceptors by cold stress, which has been shown to elevate the synthesis of anti-inflammatory cytokines including IL-1ra [48], which in turn balances and moderates the synthesis of IL-1β and then the magnitude of CRP release due to a dampened inflammatory response and faster repair of damaged muscle cells [41].

The investigations of the series of cryosessions have revealed the increases in anti-inflammatory IL-10 level in male athletes [16, 17, 43, 45] together with the decrease in proinflammatory IL-1α [45], IL-1β [43] or IL-2 and IL-8 and soluble intercellular adhesion molecule-1 (sICAM-1) expression [17]. It is worth mentioning that IL-10 is also involved in tendon remodeling [42, 49]. Ziemann et al. [2] additionally indicated the decrease in proinflammatory TNFα in professional tennis players after 10 cryosessions during 5 days. Interestingly, 3 of abovementioned studies [2, 16, 45] indicated also the increase in IL-6, pleiotropic, but generally considered as proinflammatory cytokine. Recently, according to the concept of myokines, the release of IL-6 by the muscles [50–52] has been considered as an important factor regulating metabolism and stimulating the regenerative and proliferative processes of the satellite cells. The authors [51] postulated that the exposure to an extremely low temperature might have triggered muscle shivering, resulting in an increase in IL-6. It has been also proved that IL-6 stimulates the secretion of further cytokines including IL-1ra and IL-10 [50, 51, 53] and may inhibit IL-1 and TNFα production [15], promoting the anti-inflammatory state after strenuous exercise, which has been demonstrated in human but also in endurance horses [54].

After longer therapy, including 20 cryosessions, the anti-inflammatory pattern involved the lower levels of IL-1β and also IL-6 in professional volleyball players undergoing cryostimulation, compared with untreated controls [39]. Additionally, the same amount of cryosessions produced marked decrease in α1-antitrypsin activity in elite kayaker women [55]. The repeated bout effect in the athletes should be interpreted in the context of the exercise applied. It has been shown that after an initial bout of damaging exercise, a subsequent bout within six weeks will not produce as much tissue damage [56] and results in a smaller inflammatory response [14].

Taking into account the experimental protocols, it can be postulated that randomized crossover trials, like the Pournot’s et al. [41] with two- and three-week washout periods have some anti-inflammatory effects, but the continuous therapy, including 10 or preferably more sessions, produce changes depending also on the different character of the exercise-induced inflammatory response.

Anti-inflammatory effect of extreme low temperature has also been studied in the patients with chronic polyarthritis and rheumatoid arthritis [57]. Single sessions resulted in the decrease of IL-6 and increase in IL-2 concentrations, which both return to pretreatment baseline within 3 h. Two weeks long therapy produced the decreases in proinflammatory IL-1β and IL-6, however, comparable with the results obtained in the control group undergoing traditional rehabilitation [57].

### Beneficial effects of local cold therapy in horses

In the horses, so far, only local therapy is used and so that only the effects of local procedures have been studied, mainly for the prophylaxis and treatment of laminitis. It is generally accepted that the therapeutic cooling prevents or inhibits inflammation, which has been confirmed clinically and recently, the mechanisms of this phenomenon have been investigated.

The beneficial effect of prophylactic digital cryotherapy (ICE) has been confirmed in the retrospective study, involving 130 equine patients diagnosed with colitis with evidence of systemic inflammatory response in the years 2002–2012. The procedure involved submerging the distal limb up to metacarpal phalangeal joint in ice for a minimum 48 h. The use of ICE has been shown to reduce the incidence of clinical laminitis (33% in non ICE treated vs.10% in ICE treated). Treated horses had 10 times less odds of developing laminitis compared with the animals that did not undergo this procedure. Thus, local cold therapy has been suggested as an effective prophylactic strategy for the prevention of laminitis in horses with colitis [11].

The mechanism by which cold therapy exerts its protective effect is yet not clearly understood [8, 11]. The protective vasoconstriction, impeding the delivery of “laminitis trigger factors” to the lamina, reduced metabolism that inhibit inflammatory signaling or diminished production of proteinases have been considered the most commonly [4, 8]. The anti-inflammatory effects of cold therapy have been studied in oligofructose (OF)-induced laminitis, the experimental model of sepsis-related laminitis (SRL) [5, 8–10, 12, 58] on the basis of histological examination of lamellar sections [6, 9, 12] or the lamellar expression of inflammatory factors [8, 10, 58].

Histological findings indicated that cold therapy used either as prophylaxis - cold water bath applied immediately after OF administration, for 72 h [6] or therapeutically - rubber boot filled with ice, applied after first recognition of Obel grade 2 lameness [9] for 36 h, reduced the grade of lamellar pathology. Both abovementioned studies used the same histological score based on the lesions in lamellar epidermis and the attachment to dermis [59]. In both studies, the scores were lower in the horses undergoing cold therapy, including lack of the detachment of epidermal basal cells [6] and reduced elongation of secondary lamella [6, 9]. Immunohistochemical studies revealed also the decrease in MAC387 but not CD163 positive cells in ICE treated horses [13]. MAC387 positive cells represent neutrophils and activated monocytes but are rare when compared to CD163, a marker of monocytes/macrophages both anti-inflammatory (M2) and activated (M1). As the number of CD163 cells was not changed by cold therapy, which has been documented as efficient in SRL, the authors concluded that lamellar injury was not initiated by the influx of leukocytes [13]. The examination of mitogen-activated protein kinases in cold treated horses also did not explained this problem. The concentrations of extracellular –regulated kinase (ERK) and p38 MAPK remained increased in laminitic horses, regardless the cold therapy applied. Only phospho-SAPK/JNK 1/2 increased, indicating its protective role, however, the authors were not able to determine the lamellar cell types with increased JNK activation [12].

The lamellar expression of factors involved in inflammatory response has been tested in the context of continuous digital hypothermia in a rubber boot, initiated 12 h after OF administration [8, 10] and delayed digital hypothermia, started when OG2 laminitis occurred [58]. In all mentioned studies, the expression of the same factors: proinflammatory and anti-inflammatory cytokines (IL-1β, IL-6 and IL-10), chemokines (CXCL1, CXCL6, IL-8, MCP-1 and MCP-2), adhesion molecules (ICAM-1 and E-selectin) and COX-2 have been analyzed [8, 10, 58]. After OF administration the expression of inflammatory mediators increased, however, the authors reported a little differing patterns and much different effect of cold therapy. In the horses without cold treatment both authors observed the increased expression of E-selectin, ICAM-1, CXCL1, IL-8, IL-6 and COX-2, additionally, Dern et al. [10] revealed the increases in IL-1β, MCP2, COX-1 and the decrease in CXCL-6, which decreased only in lame horses in van Eps [8] study. Cold therapy reduced the expression of inflammatory factors, however, in Dern’s [10] study only IL-6, COX-2 and lamellar leukocyte numbers decreased, while van Eps [8] reported the decreases in IL-1β, IL-6, CXCL1, IL-8 in all horses, regardless the lameness and additionally E-selectin, ICAM-1, COX-2 and CXCL6 in lame horses and the decrease in ICAM-1 and increase in IL-10 in the horses without lameness. Regardless the pattern of changes, both studies concluded that digital cold therapy started at the time point when clinical patients with sepsis are usually treated results in inhibition the inflammatory signaling [8, 10].

Dern et al. [58] examined also the effect of delayed cold therapy, applied when the lameness of one or both forelimbs occurred (OG2 laminitis) and revealed that although after OF administration the expression of several mediators increased, the cold therapy had no inhibitory effect. Thus, concluded that the protective effect of digital hypothermia initiated at the onset of clinical signs of laminitis did not result from inhibition of inflammatory pathways [58]. In the studies by Dern et al. [10, 58] the same parameters were examined, but data are presented in different ways and hardly comparable. It is also worth mentioning that van Eps [8] pointed considerable heterogeneity between horses in the lamellar expression of inflammatory mediators, which may be related to variability in susceptibility and clinical onset of the disease.

On the basis of clinical experience, the beneficial effects of cold therapy are widely accepted and also supported by scientific findings. Several methods including dry application [ice packs] or ice-water immersion are commonly used and vary in the efficacy in prophylaxis or treatment and the optimum system and protocols are not determined yet [60]. Although effective and constantly improving [61], the cooling methods may be impractical in clinical conditions, as the horse must be continuously restrained and the ice must be replenished every 1–2 h or the water must circulate through a special refrigeration system [60]. Thus, PBC, which allows much more lower temperature and time of the procedure may be considered. The main issue that has to be determined is the precise indication for this form of cold therapy. Laminitis often results from generalized “proinflammatory state”, so the prophylactic use of cryosauna may sound promising.

### Cryochamber and cryosauna

While cryochambers and cryosaunas for humans are pretty common, in veterinary medicine currently only cryosaunas may be used successfully. Temperatures inside cryodevices reach from − 110 °C to − 190 °C. Generally, there are two kinds of cooling system. First kind uses cooled nitrogen injected directly into the chamber, while the second contains nitrogen inside its walls, which seems to be safer, however, when cooling process is performed securely, dangerous situation are not likely to occur. Cryochamber for human is usually large enough for 4 or even 5 individuals. Commonly the adaptive period is necessary. This includes walking inside the chamber which is cooled from − 10 °C to − 60 °C, and after that the factual cryostimulation procedure begins. Procedure obligates subjects to move themselves during the treatment to retain the blood circulation and wear a mask to protect from breathing cold air. All these conditions are hardly applicable for horses so that only cryosaunas are used. Cryosauna is an opened tank filled with nitrogen, which directly contacts the patient’s body. The patient can breathe normally, as the head and neck are above cryosauna and so that nitrogen level. Cryosaunas do not require much maintenance and may be moved from one place to another, however, the safety rules governing the transport and storage of nitrogen pose the limitation [1]. Therapy in cryochamber and cryosauna generally have the same indications, provide similar responses and similar effects may be expected [62].

Cryogenic equipment suited for horses was introduced to the market few years ago, although it is still very rare. The Space Cabin Horse Cryosauna, produced in Ukraine allows for a range of temperature between − 110 °C and − 160 °C. For one treatment the cabin requires 20–30 kg of nitrogen and the power of 2,5 kW. The whole procedure lasts up to 5 min. The device is appropriate for one horse of regular size and the producer recommends it for the treatment of horses suffering from muscle damage, but no results confirming the efficacy of the device are provided [63]. Another example of cryosauna dedicated for horses is made in Great Britain by MAXimus s.c. Their device allows to reach the temperature − 160 °C and whole procedure takes 2–3 min. They claim that their treatment is comfortable for horses due to using a dry cooling substance. This cryosauna is recommended for horses with muscle damage, flexor trauma, hoof and coronary joint inflammation and many more, while contraindications are anaemia, blood flow disorders, recurrent airway obstruction disease and dyspnea. Again no research has been done to legitimate their statement [64].

In Poland, mobile container cryosauna has been introduced by two companies (ScreenLed and Stan-Mar). This device uses cooled nitrogen or is cooled by the chiller and allows to reach the temperature − 140 °C to − 160 °C. The procedure requires cooling the device for about 2 min and then therapeutic cryosession lasting 2–3 min may start. The temperature must be set up for individual patient, cannot be changed during procedure and is controlled by 3 sensors, placed 10 cm, 60 cm and 120 cm above the ground. The unique technology, patented by Stan-Mar, involves the circulation of cooled air, forced by the ventilator which allows to obtain the equally low temperature in cryosauna. This increases the effectiveness of cryotherapy, because the optimal therapeutic temperature (− 120 °C to − 140 °C) can be constantly applied to the patient for 2–5 min after preliminary cooling of cryosauna.

### Preparation the horse for the WBC treatment

Preparing the horse for the procedure seems quite easy. The most important condition is that the horse must be unshod and dry. If the treated animal is sensitive to noise, the ears should be covered with earmuffs.

The procedure starts with cooling cryosauna to at least − 40 °C. Horses usually walk inside without difficulty, because the whole equipment looks like a trailer. Some horses, however, needs more time and more attention to get used to the device. The treatment is painless, therefore if the horse once managed to get inside, it makes further therapy easier. While the animal is inside, nitrogen is released and the temperature quickly drops to about − 160 °C. Whole process takes less than 5 min. After the procedure horses should walk for at least 15 min to restore proper blood circulation in the limbs.

The main problem that has to be resolved is the safe and effective protocols (time and temperature) for cryosauna therapy in horses. Human studies have shown that 3 min exposure was safe and the most efficient in reducing skin temperature, when compared with 1 and 2 min exposure [65], muscular temperature was significantly decreased even 1 h after exposure [1, 66], however, large variations in skin (5.6 °C to 19.5 °C) and even core (0.05 °C to 0.8 °C) temperature have been reported depending on the protocol, the device used and the measurement location [1]. In the horses the different results may be expected due to the anatomical differences between horse and human. Thus, human data cannot be directly extrapolated to horses. Additionally, more variations may occur in the limbs due to large differences in the muscle/bone proportion in particular parts of the limbs.

In human, different protocols are recommended for therapeutic and prophylactic use. In the patients with existing pathologies including rheumatoid arthritis, fibromyalgia, multiple sclerosis and ankylosing spondylosis, the protocols must include at least ten 3 –min cryosessions, one per day, with greater effects when temperatures lower than − 60 °C are used. Generally ten to twenty 3 –min sessions are recommended. The following WBC and PBC exposures have been proposed in orthopedic and neurological diseases: for rheumatoid arthritis patients: 10 to 20 sessions, one 3-min session per day, temperature − 60 °C to − 110 °C; for fibromyalgia: 5 sessions, one 3-min session per day at − 140 °C; for multiple sclerosis 10 sessions, one 3-min session per day at − 120 °C to − 130 °C; for ankylosing spondylosis 10 sessions, one per day, 30 s at − 60 °C and then 3 min at − 120 °C; for spinal pain syndromes and peripheral joint diseases 10 session, 1-min and 2-min for the first two sessions and then 3-min exposure at − 130 °C [1].

In the athletes, even single 2 or 3 min exposure seems to have prophylactic effects. Anti-inflammatory reaction is induced when cryosessions is applied after exercise and reduce exercise-induced oxidative stress when applied before exercise. The relief of exercise-induced muscular pain was obtained after one to six 3-min exposures [1]. This seems promising for horses, as equine athletes are at high risk for getting an injury during training or competition [67, 68], particularly (43%) the damage of soft tissue structures of superficial digital flexor tendon and the suspensory ligament [68]. Thus, extremely low temperature applied to the limbs in cryosauna may similarly as in human be applied therapeutically, to shorten the recovery period after injury [14, 69] as well as prophylactically, to improve the muscular tiredness, pain and well-being after exercises [70].

## Conclusions

Clinical observations and scientific studies in human have confirmed that WBC and PBC have similar effects, are safe and effective in certain pathologies and as a support of training in elite athletes. The results from human studies and known effects of local cold therapies in horses encourage to introduce these cryotechniques to equine practice. Due to technical conditions, only cryosauna (for PBC) may currently be considered for horses and several such devices have already been introduced to European market. Up to now, neither clinical nor laboratory studies measuring the effect of cryosessions in horses are available, but some opinions sound promising. The main limitation is the lack of cryotherapy protocols for horses, however, many therapeutic procedures have already been adapted from human therapy and resulted in clinical improvement. 2 or 3-min sessions recommended for human seems proper also for horses, but this still has to be confirmed.

## Abbreviations

CRP: C-reactive protein
ERK: Extracellular –regulated kinase
ICE: Prophylactic digital cryotherapy
IL: Interleukin
OF: Oligofructose
PBC: Partial body cryotherapy
sICAM-1: Soluble intercellular adhesion molecule-1
SRL: Sepsis-related laminitis
TNF-α: Tumor necrosis factor α
WBC: Whole body cryotherapy
